# Engineered disorder and light propagation in a planar photonic glass

**DOI:** 10.1038/srep27264

**Published:** 2016-06-09

**Authors:** Sergei G. Romanov, Sergej Orlov, Daniel Ploss, Clemens K. Weiss, Nicolas Vogel, Ulf Peschel

**Affiliations:** 1 Institute of Particle Technology, Friedrich-Alexander University Erlangen-Nürnberg, Haberstr. 9a, 91058 Erlangen, Germany; 2 Interdisciplinary Center for Functional Particle Systems (FPS), Friedrich-Alexander-Universität Erlangen-Nürnberg (FAU), Haberstraße 9a, 91058 Erlangen, Germany; 3 Ioffe Physical Technical Institute, 194021, Politekhnicheskaya ul., 26, St. Petersburg, Russia; 4 Center for Physical Sciences and Technology, Savanoriu Ave., 231, LT-02300, Vilnius, Lithuania; 5 Institute of Optics, Information and Photonics, University of Erlangen-Nuremberg, Haberstr. 9a, 91058 Erlangen, Germany; 6 FH Bingen, Berlinstraße 109, 55411 Bingen am Rhein, Germany; 7 Institute of Solid State Theory and Optics, FSU Jena, Max-Wien-Platz 1, 07743 Jena, Germany

## Abstract

The interaction of light with matter strongly depends on the structure of the latter at wavelength scale. Ordered systems interact with light via collective modes, giving rise to diffraction. In contrast, completely disordered systems are dominated by Mie resonances of individual particles and random scattering. However, less clear is the transition regime in between these two extremes, where diffraction, Mie resonances and near-field interaction between individual scatterers interplay. Here, we probe this transitional regime by creating colloidal crystals with controlled disorder from two-dimensional self-assembly of bidisperse spheres. Choosing the particle size in a way that the small particles are transparent in the spectral region of interest enables us to probe in detail the effect of increasing positional disorder on the optical properties of the large spheres. With increasing disorder a transition from a collective optical response characterized by diffractive resonances to single particles scattering represented by Mie resonances occurs. In between these extremes, we identify an intermediate, hopping-like light transport regime mediated by resonant interactions between individual spheres. These results suggest that different levels of disorder, characterized not only by absence of long range order but also by differences in short-range correlation and interparticle distance, exist in colloidal glasses.

One of the most important goals of material science in contemporary photonics is developing materials with manageable light-matter interaction. The self-assembly of wavelength-scale colloidal building blocks is an attractive approach for the design of such photonic materials since they enable engineering of photonic properties in an experimentally simple and large-area approach^1^,^2^. The application area of such materials spans from photovoltaic devices^3^ and lasers^4^ to sensors^5^ and structural coloration^6^.

Conceptually, nanostructured photonic materials can be divided into either ordered or amorphous materials^7^,^8^. Ordered colloidal photonic crystals are characterized by long-range order of the individual scattering elements^1^ resulting in a periodic lattice. This order gives rise to quasi-ballistic propagation of light^9^ through the crystal and to the formation of noticeable diffractive resonances^10^,^11^, visible for certain frequencies and angles of incidence as spectrally narrow dips in transmission.

Colloidal photonic glasses have emerged as a completely new and thus very interesting class of disordered optical materials^12^. Similar to their crystalline counterparts they consist of monodisperse colloidal particles, but lack long-range periodicity. By introducing disorder, the typical spectral features of photonic crystals, which are based on the collective interaction between all the scatterers, fade away and are replaced by an optical response which is merely based on that of single particles: Diffraction resonances disappear completely in photonic glasses, while Mie resonances of individual spheres become the dominating optical effect^13^. For increasing disorder ballistic light transport turns over into diffusion^14^, and the interaction with light is restricted to only a few individual particles. One expects light transport to depend critically on the ratio between the correlation length of the disordered crystal and the extension of the near field around the individual spheres. Minute changes of the intersphere distance may affect and modify in-plane light transport and the dwell time of light at individual spheres, thus changing the strength of light-matter interaction^15^.

However, light propagation in the interesting transition region between perfect order and complete disorder has been rarely at the focus of research studies as it is challenging to introduce positional disorder into colloidal systems in a uniform and controlled manner. Structural stability of three-dimensional photonic glasses requires individual scatterers to be touching, impeding the realization of free and controlled variation of interparticle distances.

In contrast, non-closed packed ensembles of spheres can be easily realized on a substrate, allowing to precisely engineer the positional disorder via adjustment of interparticle distances without being bound by stability issues as in the case of three-dimensional glasses. Here, we study in detail the light propagation in two-dimensional colloidal photonic materials for varying the positional disorder. We assemble highly ordered, colloidal monolayers of 1 μm particles and systematically introduce disorder by mixing in a second population of smaller particles, with number ratios chosen to compromise the order of the large particles^16^. The small particles thus act as a randomizing spacer and are chosen to be resonance-less (i.e., transparent) in the spectral region of interest, enabling us to selectively probe the optical properties of an ensemble consisting of the large spheres. With increasing amount of small particles, we first compromise the long-range order of the scattering particles, creating photonic glasses with scattering particles in close proximity. Subsequently, we increase the distance between the individual scattering elements from close contact to larger separation, thus creating individual and isolated scatterers. This is accompanied by a continuous decrease of the correlation length of the lattice, *i.e*. an increase in disorder.

The optical response of planar two-dimensional photonic crystals is dominated by sharp angular and wavelength dependent dips in transmission caused by diffractive resonances^17^. At such a resonance light is coupled by one of the diffraction orders of the effective grating formed by the periodic lattice into the photonic crystal slab. The latter acts as an effective waveguide allowing for a kind of ballistic propagation of light along the crystal film^18^. Light propagating in this crystal film is finally lost to the substrate by evanescent coupling or interferes with the reflected and transmitted light if coupled back to the initial direction of propagation. This whole process is very sensitive to disorder as both the scattering of light to higher diffraction orders and the formation of guided modes in the film require a collective response of the colloidal composite. Hence, a monolayer of colloidal spheres is perfectly suited to investigate the role of disorder in the transition from a crystal to a glass.

## Results and Discussion

### Preparation and characterization of disorder in bidisperse colloidal monolayers

In order to tailor disorder in colloidal monolayers we assembled bidisperse polystyrene colloids of nominal diameters D_L_ = 1060 nm and D_S_ = 300 nm with increasing fraction of small particles at the air/water interface and transferred them to a solid glass substrate^16^,^19^. Progressively disordered monolayers will be further referred to as MB1 ÷ MB3 (monolayer – binary) samples, respectively, to distinguish them from a monolayer crystal of large monodisperse beads which we refer to as ML (monolayer). We chose the size of the small particles in a way that they do not feature any resonances in the wavelength region of interests and thus merely act as transparent, physical spacers for the large spheres, which form the dominant scattering units.

**Figure 1:**
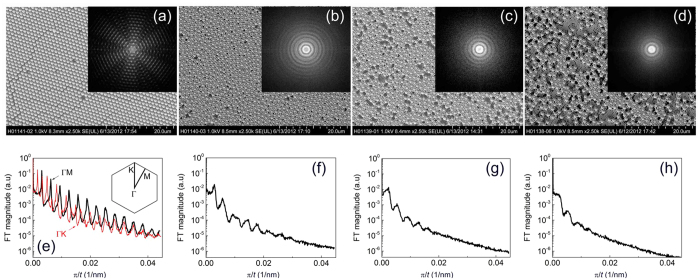
Characterization of order and disorder in binary colloidal monolayers. Scanning electron micrographs of (**a–d**) ML, MB1, MB2 and MB3 samples. Insets show the 2D Fourier transforms of the corresponding arrays of large colloids. (**e–h**) Fourier transform (maximum normalized to 1) as a function of distance in reciprocal space. In case of the ML sample (panel (**e**)), plots correspond to cross-sections of the 2D pattern along ΓM and ΓK axes of the reciprocal hexagonal lattice as indicated in the inset to this panel. In case of panels (**f–h**) we took the angular average.

In Fig. 1 we illustrate the evolution of a two-dimensional colloidal photonic crystal with long-range order towards a disordered photonic glass by increasing interparticle distances. To quantify the effective ordering and the distance between spheres we analyzed the 2D Fourier transform (FT) patterns of the SEM images displayed in Fig. 1a–d (shown as insets), taking into account only the positions of the large spheres. The FT pattern of the ordered monolayer (ML) reveals its hexagonal symmetry, whereas in binary colloid monolayers (MB1-MB3) the FT pattern is transformed to an ever reducing number of concentric circles, indicating loss of long range order. Still these rings indicate some short-range order and correlated average distances between particles. The higher the number of rings, the longer the correlation extends in space. In the least disordered sample MB1, the number of circles substantially exceeds that of samples with increasing fraction of small spheres in the suspension (MB2-MB3, from left to right). In spite of the apparent randomization in the array of large spheres, a residual correlation between spheres persists. This correlation extends over approximately 8 particles (MB1, Fig. 1f), approx. 5 particles (MB2, Fig. 1g) to only 3 particles for the most disordered sample (MB3, Fig. 1h). The distance between the rings of the power spectrum of the Fourier transform corresponds to the effective separation of large spheres in the SEM images. The latter increases from ~1000 nm in ML sample to ~1110 nm, 1420 nm and 1630 nm in binary monolayers.

**Figure 2:**
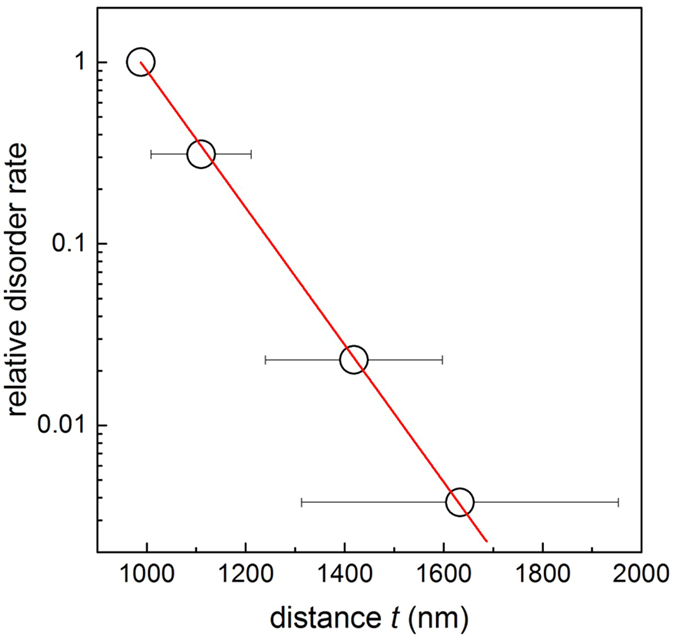
Quantification of order in binary colloidal monolayers. The diagram shows the ensemble ordering, calculated from cross-correlation functions of the SEM images shown in Fig. 1 as a function of the mean intersphere distance. The order rate of the ML sample is chosen to be equal 1. Error bars indicate the width of the respective peaks in the Fourier spectra. The red line shows the exponential decay fit to experimental data.

In our samples order is directly related to the sphere spacing. We quantified the residual amount of ordering by evaluating the cross-correlation functions between two SEM images of each sample taken at different positions. In Fig. 2, we plot the ratio of the ensemble-averaged magnitude of this cross-correlation function of the disordered and of the ordered ensembles against the effective sphere separation. The plot reveals an exponential decrease of the global ordering with increasing sphere separation by a maximum factor of ~250 (Fig. 2). From numerical fitting we derived that the exponential decay function constructed on the basis of diameters of bidispersed colloid 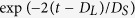, where 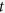 is the mean distance between sphere centers, approximates the experimental points well. Noteworthy, this functional form needs to be verified with other 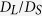 ratios, which is beyond the scope of this work. Hence, ordering can be predictably and precisely tuned by several orders of magnitude by varying the partial concentration of small spheres in the binary colloidal monolayers.

### Characterization of light transmission through colloidal monolayers with increasing degrees of disorder

**Figure 3:**
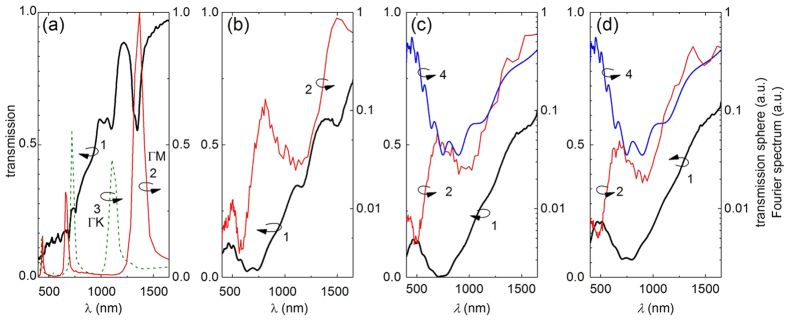
Influence of order on transmission spectra. Comparison of transmission spectra (black lines, labelled 1, taken at *θ* = *0*^*o*^) of slab two-dimensional colloidal photonic structures with the average interparticle distance obtained from Fourier transform spectra of Fig. 1 (red lines, labelled 2). (**a**) Ordered colloidal monolayer ML. Here we used two Fourier transforms of the SEM image in Fig. 1a taken along the ΓM and ΓK directions of the lattice Brillouin zone as indicated in the inset to Fig. 1e, which are labelled 2 and 3, respectively. (**b–d**) The same as (**a**) for the monolayers with increasing disorder, MB1–MB3 sample, respectively (curves 1 and 2). Curve (4) – the calculated transmission spectrum of 1063 nm spheres in the air.

After having characterized the morphology of our samples we analyzed their optical properties by evaluating the respective transmission spectra. As expected, the transmission spectrum of the ordered monolayer displayed in Fig. 3a shows pronounced minima (curve 1, black line), the spectral positions of which are well correlated with length scales of the Fourier spectrum of the SEM image (curve 2, red line,) or with the inverse of the particle separation along typical symmetry axis of the lattice (curve 3, dotted line).

This strong correlation between interparticle distances and spectral positions of intensity minima vanishes with increasing disorder (Fig. 3, panels a to d). In the same way as collective effects responsible for grating resonances diminish, the optical properties of large individual spheres gain relevance. The transmission spectrum of the disordered samples (Fig. 3b–d, curves 1, bold black line) is significantly modulated by a series of resonances, which are similar in all disordered samples. The striking similarity of these resonances to the calculated spectrum of Mie resonances of the 1060 nm diameter polystyrene sphere immersed in air is indicative for assigning them to individual spheres (compare curves 1 and 4, Fig. 3c,d).

**Figure 4:**
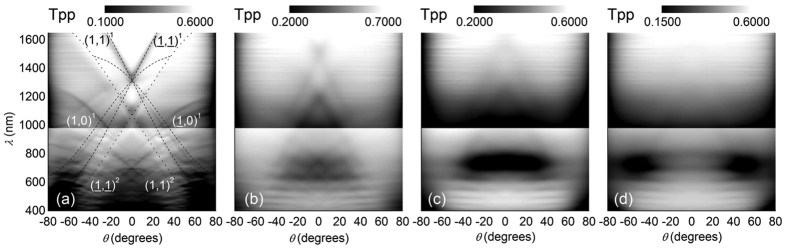
Loss of ballistic light transport in quasi guided modes with increasing disorder. (**a–d**) Angle- and frequency resolved transmission patterns of ML, MB1, MB2 and MB3 monolayers. The lines in panel (**a**) show the calculated dispersion of diffraction orders caused by quasi-guided modes, which are labelled by the respective vectors of the reciprocal lattice and the index referring to the number of the group. Only selected modes are plotted. The stitching of patterns at 980 nm is due to the change of the spectrometer. The transmission magnitudes of two parts are not aligned in order to increase the visibility of spectral details. The logarithmic grey scale is quoted for the long wavelength parts. With increasing disorder, the signatures of collective, diffraction resonances are increasingly suppressed.

Angle-resolved transmission spectra tell even more about light transport in the investigated monolayers as the width of their angular-dependent narrow minima is inverse to the propagation length of respective modes guided in the slab (Fig. 4a). Introducing disorder in the monolayer leads to a broadening of respective resonances demonstrating a reduced range of ballistic transport in the layer (see Fig. 4b,c). The gradual loss of long range order also weakens the diffraction efficiency of the corrugated slab making an excitation of modes propagating along the film unlikely, and thus, reducing the contrast of respective resonances in proportion to reducing long range ordering (Fig. 1). Finally all collective effects causing diffraction resonances disappear, only to be replaced by Mie resonances of individual spheres in the mostly disordered sample (see Fig. 4d). Hence, the balance between collective light transfer and single scattering can be tuned by adjusting the order rate.

**Figure 5:**
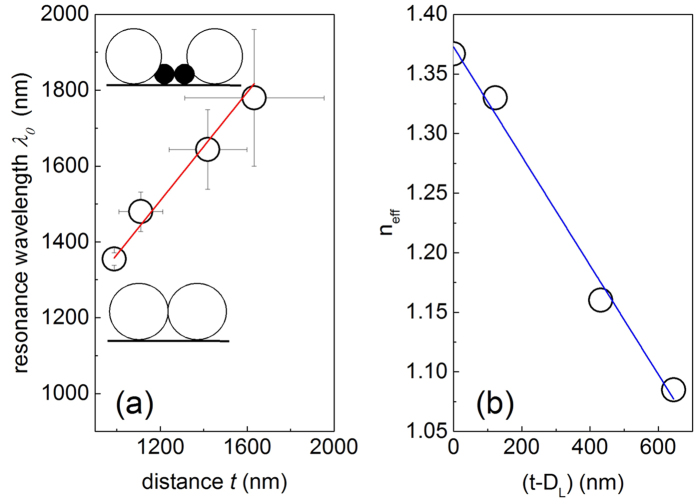
Shift of diffraction resonance with increasing disorder. (**a**) Positions of the long wavelength diffraction resonance against the intersphere distance. Error bars indicate the width of the respective peaks in the Fourier and transmission spectra. Schematics illustrate sphere arrangements in monolayers. (**b**) The effective refractive index extracted from diffraction resonances (circles). Straight lines are the linear approximations to these data as discussed in the text.

While width and contrast of diffraction resonances critically depend on the strength of disorder, also their spectral positions shift. To determine the resonance wavelength, *λ*_0_, for the disordered samples we extrapolated the traces of minima in angle-dependent transmission patterns (see Fig. 4b–d) towards their intersection at *θ* = *0*^*o*^. According to the numerical fitting of experimental points, the resonance wavelength changes approximately linearly with the sphere separation as 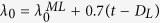 (see Fig. 5a). The diffraction resonance wavelength in monolayers corresponds to the optical path length between large spheres, which is a product of the intersphere distance and the effective index of refraction, *n*_*eff*_. It occurs that 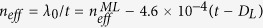 drops linearly with increasing separation of large spheres from the value of the ordered monolayer 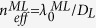 as shown in Fig. 5b.

The above-mentioned independence of Mie-like resonances of large spheres on the inter-sphere separation is confirmed by angle-resolved transmission patterns within the experimental accuracy (see Fig. 4b–d). Provisionally, transmission spectra of monolayers show signatures of an additive character, i.e., the collective resonances of quasi-ordered sphere clusters are superimposed with individual resonances of loosely positioned large spheres. The Mie-like resonances of the latter spheres remain unaffected because these spheres are not surrounded by small ones and these resonances appear in the spectral range where the sphere diameter exceeds the wavelength of the incident light. This observation motivates a need for the microscopic modelling of light transport in disordered monolayers.

### Characterization of light propagation within colloidal monolayers with increasing degrees of disorder

**Figure 6:**
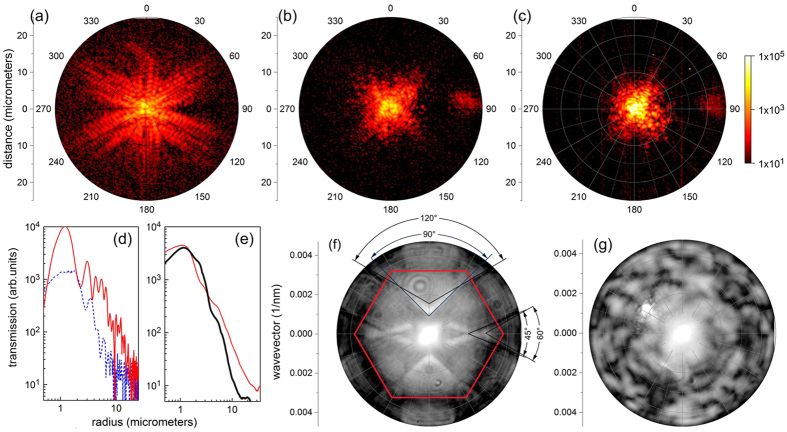
Direct visualization of in-plane light transport in ordered and disordered colloidal monolayers. (**a–c**) Images of the cross-polarized backscattered light intensity from the ordered monolayer (ML) and from two different points of the most disordered binary monolayer (MB3) sample, respectively The excitation wavelength was 1200 nm and the beam was focused on a single sphere in the center of the image using the objective of NA = 0.9. (**d**) The decay of the backscattered light intensity with increasing distance from the excitation spot in the ordered monolayer (ML) sample, extracted from panel (**a**) along a crystal axis (red, solid line, taken along the 60^o^ direction) and beside any crystal axis (dashed blue line) taken along the 90^o^ direction). (**e**) The azimuth-averaged decay of captured light in the ML (red, thin line) and MB3 (black, thick line) samples. (**f,g**) Fourier plane images of the light scattered by ordered (ML) and disordered (MB3) samples in co-polarized light, respectively, obtained at the same points as scattered light patterns in panels (**a,b**). The red hexagon in panel (**f**) shows the Brillouin zone of the hexagonal lattice.

To this point, we only investigated disorder-induced changes of the spectral properties of colloidal systems, but did not measure light transport in the layers directly. To understand in-plane light propagation in detail, we focused a linearly polarized laser beam on a single sphere of the monolayer and detected the backscattered light in cross polarization mode (Fig. 6a–e). As the beam focus was small enough to illuminate just a single sphere and because the objective imaged the surface of the sample, all light observed outside the cross section of the incident beam must have first to propagate in the layer to be scattered out at the point of observation thus directly visualizing the leaky light transport in the sample.

Light transport in the ordered monolayer was found to be significantly anisotropic and predominantly oriented along chains of touching spheres in the hexagonal crystal (see Fig. 6a). It can be traced at least 25 spheres away from the excitation spot. Detected far-field pattern observed in the Fourier plane of the objective (see Fig. 6f) reflect the symmetry of the respective lattice. In case of the two-dimensional photonic crystal (Fig. 6f) they also display the occupation of the iso-frequency surfaces of the band structure of modes guided in the crystal by the injected light. Consequently a 6 fold symmetry due to the lattice overlaid by a twofold one imposed by the linear polarization of the exciting beam is observed (see angular sectors in Fig. 6f).

In the case of the most disordered sample, where collective interaction between different spheres seems to be absent (see Fig. 4d), we still observe anisotropic light transport (see Fig. 6b,c), but without a noticeable symmetry. Due to enhanced out-of-plane scattering the intensity decays faster than in case of the ordered sample and after a propagation along a path of ~10 spheres the strength of the scattered light has reached the background level. The decay of the intensity of the scattered light with distance follows a power law in both ordered and disordered arrays (Fig. 6d,e). In the ordered hexagonal lattice of spheres the decay is slightly anisotropic and decreases as *r*^−2^ vs *r*^−2.8^ for two principal lattice directions, with the slower decay along the chain of touching spheres within the crystalline lattice. Averaging over all azimuth orientations gives rise to a decay like *r*^−2.3^. The intensity of light scattered from the amorphous monolayer decays much faster, but still in a polynomial fashion with an exponent between −3 and −4.5 depending on the point of excitation. The respective Fourier plane image confirms the absence of extended collective modes for the in-plane travelling light (Fig. 6g). The similar behavior applies to other loss images acquired in the range 1000–1700 nm.

### Simulations of energy flux

**Figure 7:**
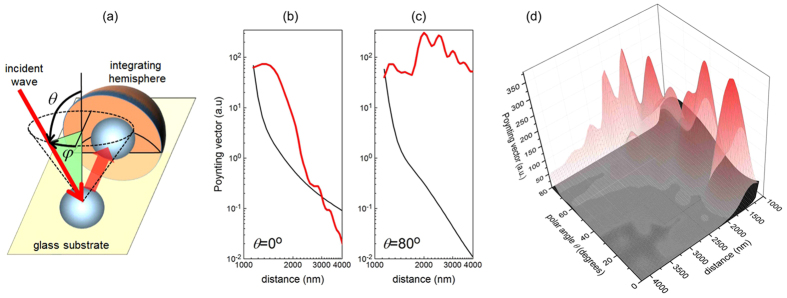
Energy flux between two spheres placed on a glass substrate and separated at wavelength-scale distances. (**a**) Schematics of the calculated model, two spheres in the air laying of a glass substrate. (**b,c**) The dependence of the transferred amount of power (the Poynting vector) of s-polarized light between two 1068 nm spheres on the glass substrate as a function of the distance when the sphere-receptor is excited along the line connecting these spheres, λ = 1200 nm, *φ* = 0^o^ for two polar angles, θ = 0 and 80^o^. Red and black lines are calculated with and without the back-action of spheres in a dimer, respectively. (**d**) The energy flux as a function of the separation distance and the polar angle θ at λ = 1200 nm, *φ* = 0^o^.

The experimentally determined polynomial decay of light transport is supported by respective calculations based on scattering matrix theory. We first investigated the energy flux between two 1060 nm diameter polystyrene spheres laying on a glass substrate. We assumed that one of the spheres was excited by a plane wave and that in the next step the energy was transferred to the other sphere. The intensity emitted by the second sphere into free space was integrated vs the hemisphere displayed in Fig. 7a and served as a measure of the efficiency of a two stage hopping process. At normal light incidence the simulated configuration is similar to that investigated experimentally above, but with only two spheres instead of the entire ensemble. Based on our code we changed the separation between the two spheres and studied the induced variation of the power radiated by the second sphere. Surprisingly we found the power transfer between the two spheres not to decay monotonously with distance, but to show even noticeable oscillations in case of oblique incidence (see red lines in Fig. 7b,c). Only if back action between the spheres was neglected in the simulation the two stage hopping process decayed rapidly as a function of sphere separation with an *r*^−5^ dependence (black lines in Fig. 7b,c). Hence, collective effects between the spheres cannot be neglected as it may increase the power transfer by several orders of magnitude. Resonant enhancement and pronounced distant dependent oscillations are even more pronounced in case of oblique incidence, i.e. for an excitation being merely directed along the layer (see Fig. 7d). As the excitation of a single sphere (see Fig. 6) was performed with a high NA objective (NA = 0.9) also those angular components play an important role.

**Figure 8:**
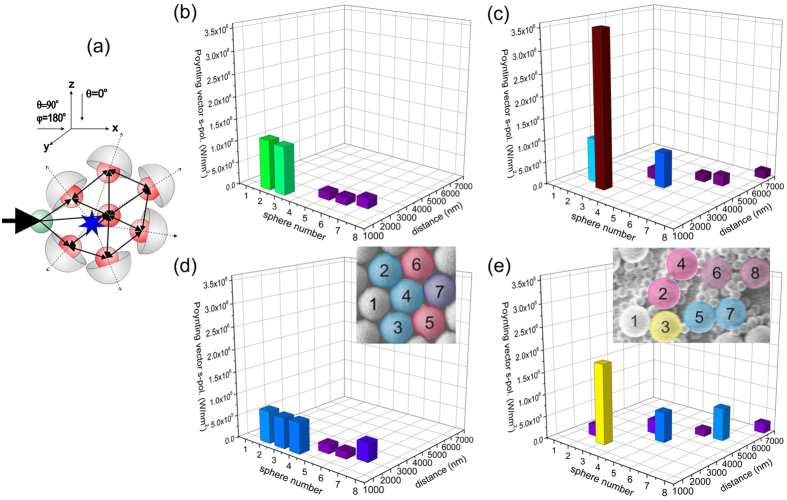
Energy flux radiated by ordered and disordered clusters of spheres placed on a glass substrate. (**a**) Schematics of the cluster. The s-polarized incident plane wave excites sphere ^#^1, from which the flux enters the cluster by means of the nearest neighbor interactions. The star indicates the position of the center of mass and arrows the pair interactions. (**b,c**) Radiated fluxes by spheres of the ordered and disordered clusters as a function of the distance from sphere ^#^1.The flux radiated by each of the outer spheres is integrated vs. a hemispheres oriented along the line connecting the center of mass and the sphere center. (**d,e**) The flux radiated by each of the outer spheres is integrated vs. a hemispheres oriented normally to the glass substrate. Insets show SEM images of modelled clusters with enumerated spheres. The color of the spheres is chosen to match with the intensity of the radiated flux normal to the monolayer plane.

To get better insight into more involved hopping processes we performed further simulations based on scattering matrix theory and on SEM images of our samples. We focused on higher order hopping processes, i.e., we calculated the energy transferred from an excited sphere #1 into the cluster of spheres and registered the power radiated by each of the outer spheres into the far-field. Only pair interactions between the nearest neighbors were taken into account as shown in Fig. 8a. To highlight energy transport along the layer, the hemispheres that integrate the radiation were oriented away from the cluster’s center of mass. To adjust the simulations to the experimental conditions, we integrated the outgoing flux over the whole range of angles of incidence within the numerical aperture of the objective (NA = 0.9) and over all azimuth directions. As a consequence sharp resonances may have smeared out, but still collective effects seem to be present similar as in the two stage hopping process. According to our simulations the efficiency of the energy transfer critically depends on the actual configuration and does not decay monotonically with increasing distance from the point of excitation. In case of the ordered cluster (Fig. 8b) the highest power is as expected radiated by the nearest neighbors of sphere #1, hence by spheres #2 and #3. But the next largest flux is emitted by sphere #7, which is further away from sphere #1 than spheres #5 and #6. Also the power radiated by spheres of the irregular cluster decays with growing separation from sphere #1, but again not monotonously (Fig. 8c). For example spheres #6 and #7 emit substantially different power although being almost equally separated from #1. As the simulations show, power transfer rates in the ordered and disordered cluster do not differ considerably, but are both seemingly affected by resonances. In particular in case of the disordered cluster isolated dimers may by chance establish a very efficient power transfer similar as it was observed for the two stage hopping process. For example the power transfer between the spheres 1 and 3 in the disordered cluster is even stronger than between sphere 1 and 3 in the ordered cluster although the spacing is the same.

Since, the data in Fig. 6 represent the flux emitted out of a monolayer plane, we calculated the respective fraction of light that is radiated in air. This radiation is proportional to the captured one, moreover, the intensities of both fractions are not dramatically different (Fig. 8d,e). Thus, the measurements of losses in the air correctly estimate the flux captured in ordered and disordered monolayers. Qualitatively similar results have been obtained from simulations performed at several wavelengths across the visible and near-infrared spectra range.

## Conclusion

In summary, we prepared two-dimensional colloidal photonic glasses with tailored disorder via the self-assembly of monolayers of binary colloids. With increasing number ratio of smaller colloids, the ordering of the large particles is increasingly compromised, leading to a decrease in global order and an increase in average distance between the large scattering particles. We quantified the disorder via Fourier transform analysis of the structures. Even in absence of long range order, we still observed distinct rings in the Fourier transformed images, the number of which corresponds to the average size of regular areas expressed in sphere diameters and is reducing in our samples from about 8 to 3 with increasing disorder. Such adjustment of disorder properties is enabled by the two-dimensional nature of the crystal, with which we mitigate the necessity for touching spheres that is typically found in three-dimensional colloidal photonic glasses.

While the spectral and angular optical response of the ordered photonic crystal layer is dominated by the presence of well-defined and sharp diffraction resonances, the influence of such collective effects fades away with increasing disorder. Even if long range order is completely lost and the Fourier transform of the layer shows only about five rings we still observe traces of diffraction resonances, which have only lost most of their contrast and sharpness. In the same way as diffraction resonances disappear, Mie resonances resulting from the scattering of individual spheres gain relevance. For the most disordered layer the far-field optical response can be well described by that of single spheres placed on a transparent substrate.

The appearance of diffractive resonances is strictly related to the excitation and propagation of a quasi-guided mode in the colloidal film and is therefore a manifestation of collective interaction between the spheres resulting in a long range energy transport which is only limited by radiative coupling to the substrate and air. In contrast, single particle Mie resonances, as they determine the optical response of the most disordered sample, do not provide any indication for the existence of energy exchange between different spheres. Still such transfer processes exist in all samples. They were detected by optically exciting a single sphere of the layer by a tightly focused beam. We imaged all light, which propagated away from the point of excitation and which was scattered towards air thus providing information about light transport in the film. In all cases the observed intensity pattern decaying in a polynomial fashion as a function of distance from the excitation spot indicating a strong influence of near field coupling. Light transport was the most efficient in the ordered sample with a preferred direction along touching spheres. Compared with the regular array, the decay was faster in the most disordered sample, but still a significant power flow could be observed. A comparison with numerical simulations revealed that energy exchange in the most disordered film was dominated by a two stage hopping process from the excited sphere to a sphere emitting light to the far-field. Higher order hopping processes still seem to play a role even in the most disordered sample.

Our results indicate that different levels of disorder, characterized not only by absence of long range order but also by differences in short-range correlation and interparticle distance, can be prepared in a colloidal glass. These different disordered states show drastically different optical properties which are closely connected to the microstructure and separation of the individual scatterers.

## Methods

### Colloid synthesis and assembly

Colloidal particles were synthesized by a surfactant free emulsion polymerization using styrene and acrylic acid as co-monomers and purified by extensive dialysis and centrifugation^16^. 2D Self-assembly was performed on a Langmuir trough (KSV5000, 732 cm^2^ surface area) following a procedure described in literature^20^. In brief, binary dispersions with increasing number ratio of small particles (S, *D* = 300 nm) with respect to the number of large particles (L, *D* = 1060 nm) were mixed (S:L ratio: 0, 10:1, 15:1, 20:1) and added to the air/water interface with a pH of 6 and, after equilibrating for 5 min, compressed with a speed of 10 mm/min until the onset of collapse in the isotherm. The transfer of monolayers to solid substrates was performed by lowering the water surface until the substrate surface was covered with colloids.

### Structure analysis

2D Fourier transformation was applied to SEM images in order to visualize the repeating elements of the structure. The first maximum was used to find the intersphere distance. Two SEM images acquired from different areas of one and the same sample were used to calculate the 2D cross-correlation function. The magnitude of the area-averaged cross-correlation function was used as the measure of the array ordering degree.

### Transmission spectroscopy

Samples were illuminated by a collimated beam of linear polarized white light of 1 mm in diameter from a tungsten lamp. Transmission spectra were obtained in the zero diffraction order as a function of the incidence angle of the light beam. Collinear polarizers were placed before and after the sample. The polarization of the light that reaches the spectrometer was scrambled in order to avoid the interference with the spectrometer grating.

For the sake of comparison with experimental transmission spectra we converted the calculated extinction spectra 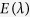 of polystyrene spheres in transmission using the expression 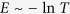.

### Imaging light propagation

Light originating from a supercontinuum light source was filtered by an acousto-optical tunable filter to achieve a bandwidth of *λ*_0_ = 5 nm in the range of 1 to 1.7 micrometers. A collimated and linearly polarized Gaussian beam was focused on the sample surface (NA = 0.9, dry objective) where it formed a diffraction-limited spot with diameter <2 μm. The backscattered light from the sample was imaged with the in-coupling objective passing a NIR-polarization filter and detected by an InGaAs CCD camera with a 150x magnification. For cross-polarized measurements the polarization filter in front of the CCD camera was set perpendicularly with respect to the polarization filter for the incoming beam, thus suppressing the back reflected light from the sample with a ratio of 1 to 10000.

### Numerical simulations

The incident electric field was first analytically expanded into electromagnetic multipoles (also called vector spherical harmonics (VSH))^21^ and then rotated using the rotation theorem for vector spherical harmonics^22^. The exciting electric field **E** can thus be expressed as


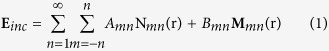


where **N**_*mn*_ and **M**_*mn*_ are regular VSH representing differently oriented electric and magnetic multipoles and the incident electric field is described by complex-valued multipole expansion coefficients *A*_*mn*_ and *B*_*mn*_ 21. This representation permits a simple treatment of scattering problems via the so-called T-matrix approach^22^, where the scattered field **E**_***sca***_ is expressed as 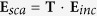, where **T** is the T-matrix of an object in the free space. For an object placed on a substrate the interaction of the object and the substrate with the incoming light field is accounted for by an effective scattering matrix **T**_eff_


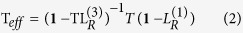


**L**_R_^(1,3)^ represents the reflection operators of the substrate (see Supplementary material in^22^,^23^). In the case of a cluster containing N spherical particles, its object T-matrix can be calculated from a T-matrix of a single scatterer **T**_sphere_ and from the relative distances **r**_ij_ (i, j = 1, …, N, i ≠ j) between particles in the cluster using vector translation theorem for VSHs^21^. The power scattered into the angles ([*θ*_1_, *θ*_2_], [*ϕ*_1_, *ϕ*_2_]) can then be expressed by


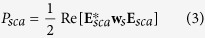


where **w**_s_ is a matrix, containing scalar products of the far-field functions of the vector spherical functions and acts as a scattering operator, see^23^,^24^ and **E**_sca_ is the field scattered by an object.

## Additional Information

**How to cite this article**: Romanov, S. G. *et al.* Engineered disorder and light propagation in a planar photonic glass. *Sci. Rep.* **6**, 27264; doi: 10.1038/srep27264 (2016).
